# Status and hotspot analysis of Qingfei Paidu Decoction for the prevention and treatment of COVID-19 based on bibliometric analysis

**DOI:** 10.3389/fphar.2024.1422773

**Published:** 2024-07-31

**Authors:** Huifang Zhang, Yang Liu, Xiyu Shang, Yuqing Cao, Jiajia Li, Guangkun Chen, Xinyu Ji, Lei Zhang, Yipin Fan, Yan Ma

**Affiliations:** ^1^ Institute of Basic Research in Clinical Medicine, China Academy of Chinese Medical Sciences, Beijing, China; ^2^ Shaanxi Academy of Traditional Chinese Medicine, Xi’an, China; ^3^ Institute of Traditional Chinese Medicine Information, Chinese Academy of Traditional Chinese Medicine, Beijing, China

**Keywords:** Qingfei Paidu Decoction, COVID-19, traditional Chinese medicine, bibliometrics analysis, CiteSpace

## Abstract

**Background:**

Qingfei Paidu Decoction (QFPDD) has played an important role in the prevention and treatment of COVID-19 infection in China. The present study aims to perform an econometric analysis and visualization of the literature on the treatment of COVID-19 with QFPDD in the Chinese databases and English databases.

**Methods:**

Six databases including such as Chinese databases CNKI, VIP, CBM, WANFANG as well as English databases PubMed, Web of Science were searched for publications related to the prevention and treatment of COVID-19 with QFPDD. The institutions, authors, keywords of each publication were cisualized using the software of CiteSpace.

**Results:**

A total of 187 literature on the prevention and treatment of novel coronavirus infection with QFPDD were included, of which 145 (77.5%) were in Chinese and 42 (22.5%) were in English. Those publications were written by 926 authors from 383 institutions. There were 78 theoretical studies (41.7%), 63 clinical studies (33.7%), and 46 basic studies (24.6%). The cooperative institutions with the core of “Institute of Basic Research in Clinical Medicine, China Academy of Chinese Medical Sciences” and “Shanghai University of Chinese Medicine Cross Academy of Science” have been formed, and two core teams with “Wang Yanping” and “Zhang Weidong” have been formed. The keyword analysis showed that the research mainly focuses on pathologic pathogenesis, clinical efficacy, adverse reactions, integrated Chinese and western medicine therapy, network pharmacology research.

**Conclusion:**

QFPDD has attracted worldwide attention, mechanism research and clinical research may become a future development trend. Therefore, in-depth basic research and clinical studies with large samples and multi-center cooperation should be carried out to provide high-level evidence-based evidence for the prevention and treatment of COVID-19 with QFPDD.

## 1 Introduction

COVID-19 stems from an infection triggered by the SARS-CoV-2 virus (Selva-Pareja et al., 2023). AS of 13 April 2024, there were more than 700 million cases, and over 7 million death cases (World Health Organization, 2024). Coronavirus disease 2019 (COVID-19) is classified as an “epidemic” according to traditional Chinese medicine (TCM). TCM has played an important role in the treatment of COVID-19 and has provided China’s solutions in the global fight against the epidemic (Fan et al., 2020a; Li and Xu, 2020; Zhao et al., 2020; Fan et al., 2020b; Zhang et al., 2021; Ma et al., 2020; Zong et al., 2022; Xiong et al., 2024). The World Health Organization issued an expert evaluation report on 31 March 2022 that recognized that TCM has played an important role in reducing the proportion of mild-to-moderate COVID-19 cases progressing into severe cases (World Health Organization, 2022). Qingfei Paidu Decoction (QFPDD) has attracted extensive attention due to its wide range of applications and remarkable clinical efficacy (Song et al., 2020). The National Administration of Traditional Chinese Medicine and the National Health Commission of the People’s Republic of China jointly issued a document on 6 February 2020 promoting the use of QFPDD as a general prescription nationwide (National Health Commission of the PRC, 2020).QFPDD is a prescription modified from ancient classical prescriptions, including Maxing Shigan Decoction, Wuling Powder, Xiaochaihu Decoction, and Shegan Mahuang Decoction, and is suitable for treating the core pathogenesis of COVID-19 (Fan et al., 2020c; Li et al., 2020; Li et al., 2021; Tong et al., 2021). In clinical practice, QFPDD has been shown to be effective at preventing the progression to severe COVID-19 (Ma et al., 2020; Shi et al., 2020; Jang et al., 2021), significantly improving clinical symptoms, shortening the time to a negative nucleic acid test (NAT), promoting the absorption of lung lesions, and lowering the mortality rate. Its efficacy is better when it is used at the early rather than late stages of disease (Fan et al., 2020a; Shi et al., 2020; Wang et al., 2021; Zhang et al., 2021; Sun et al., 2021; Yu et al., 2020; Ge et al., 2022). Systematic pharmacological studies have revealed that QFPDD exerts anti-infective, anti-inflammatory, and multi-organ protective effects through multiple targets and pathways in the treatment of COVID-19 (Peng et al., 2020). Previous studies have also shown that the leupeptin in QFPDD inhibits the replication of severe acute respiratory syndrome coronavirus 2 (SARS-CoV-2) (Fu et al., 2021). Many academic papers on the prevention and treatment of COVID-19 with QFPDD have been published that have discussed its efficacy and mechanism of action, it is suggested that QFPDD has potential in improving the treatment outcome of COVID-19, however, a systematic and comprehensive in-depth overview on QFPDD and COVID-19 is currently lacking. Therefore, an overview of the literature on the prevention and treatment of COVID-19 with QFPDD is urgently needed to provide a high-level evidence base for its use.

With studies on QFPDD for the prevention and treatment of COVID-19 infection increases and continues to expand rapidly, which brings challenge to extract and synthesize the large available existing information. Bibliometric analysis is reported to be an indispensable tool, which is a method that quantitatively describes literature published using mathematics and statistics (Atlasi et al., 2022). It is widely used to explore research hotspots and development trends in a specific field (Huang et al., 2015). We used bibliometric methods to systematically and comprehensively overview the relevant literature on QFPDD for the prevention and treatment of COVID-19. Moreover, we conducted a visual analysis to help understand the current research status, hotspots, and frontiers in China and abroad, with the aim of providing a reference and information to facilitate research on QFPDD for the prevention and treatment of COVID-19.

## 2 Materials and methods

### 2.1 Literature sources

Data were retrieved from six databases, including four Chinese databases such as China National Knowledge Infrastructure (CNKI), China Science and Technology (VIP), China Biology Medicine databases (CBM), and WANFANG, and two English databases such as PubMed and Web of Science. The Chinese databases were searched using the following strategy: Title/Keyword/Abstract = [(“清肺排毒” OR (“清肺”AND”排毒”)] AND (“汤 OR” 方 OR “颗粒” OR “合剂”) AND Topic = (“2019冠状病毒” OR “新型冠状病毒” OR “新冠肺炎” OR“2019-nCoV”OR “SARS-CoV-2” OR “Novel coronavirus” OR “nCoV” OR “Emerging Coronaviruses”OR “new coronavirus” OR “COVID-19” OR “coronavirus”). The English databases were searched using an advanced search method with the following strategy: TS = (“Qingfei Paidu decoction”OR “QFPDD”OR “QPDD” OR “qfpdt” OR “Lung Cleansing and Detoxifying Decoction” OR “Lung Cleaning and Detoxifying Decoction”) AND (“COVID-19” OR “covid 19” OR “sars cov 2” OR “coronavirus 2019” OR “coronavirus disease 2019” OR “Novel coronavirus” OR “Emerging Coronaviruses” OR “new coronavirus” OR “2019-ncov”). Literature published from 1 January 2020 to 31 March 2024 was searched.

### 2.2 Literature selection criteria

Academic literature related to QFPDD and COVID-19 was included in this study. The following items of literature were excluded: 1) literature unrelated to this research field, 2) interviews and newspaper articles, 3) reports of results and standards, 4) duplicates, and 5) literature with missing information.

### 2.3 Literature search

**FIGURE 1:**
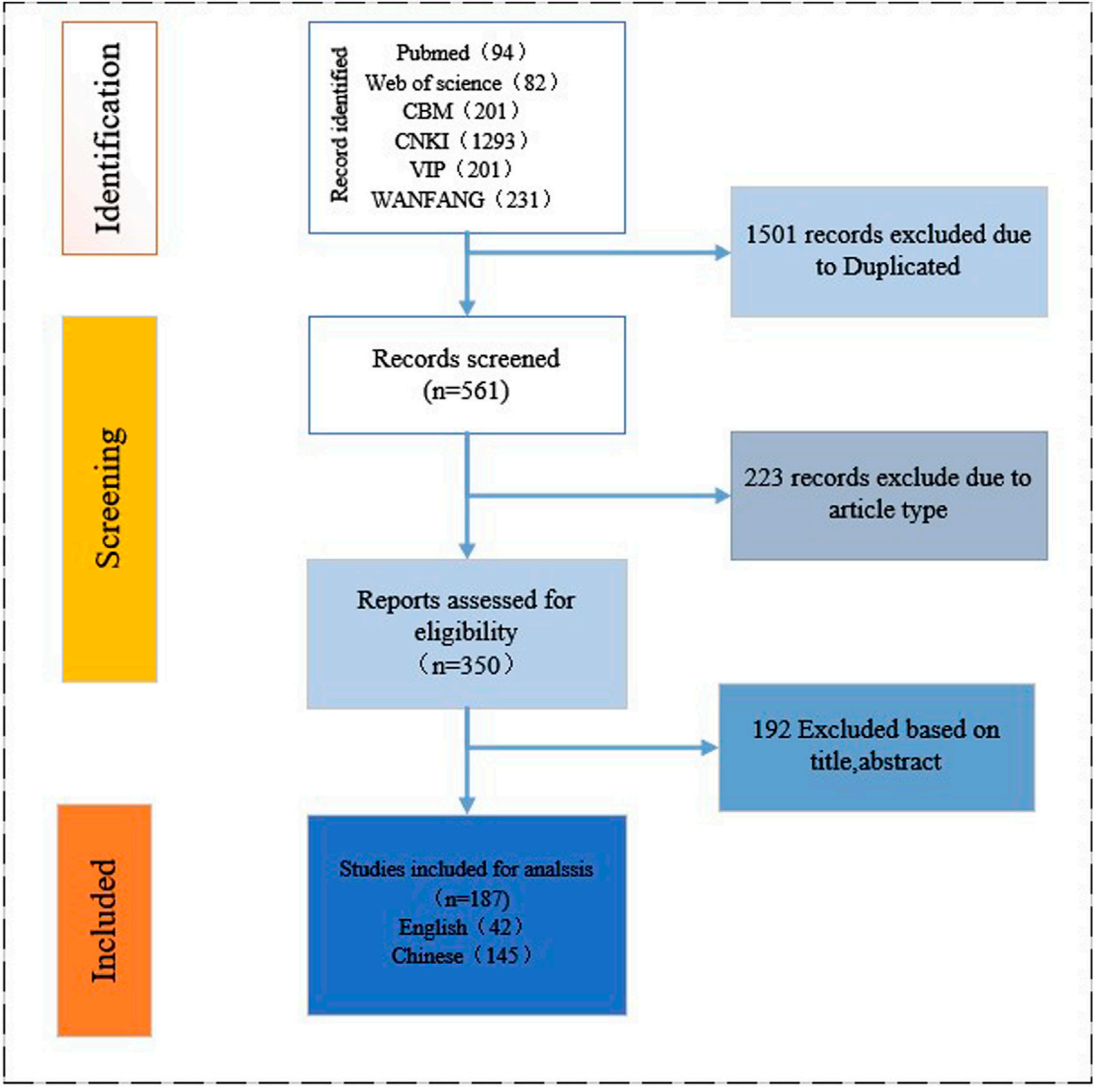
Flowchart of the study selection process.

Initially, 2,103 articles were identified, including 1,925 in Chinese (1,293 from CNKI, 202 from VIP, 201 from CBM, and 231 from WANFANG) and 176 in English (94 from PubMed and 82 from Web of Science). After excluding 1,916 articles (1,501 duplicates; 223 opinions, newspaper articles and experience summaries; and 192 unrelated articles), 145 Chinese and 42 English articles were included (Supplementary Table S1). The selection process is shown in Figure 1.

### 2.4 Literature screening and data extraction

Two researchers independently performed the literature search and data extraction, and cross-checked the results. In cases of disagreement, a third researcher assisted in the judgment. The titles, authors, institutions, journals, keywords, years of publication, and abstracts were exported from the retrieved articles for deduplication using Endnote literature management software (Clarivate, London, United Kingdom). The results were then imported into NoteExpress for the preliminary screening against the title, keyword, and abstract to exclude articles unrelated to the prevention and treatment of COVID-19 with QFPDD. A database of bibliographic information including the article titles, years of publication, journals, keywords, authors, institutions research types, research objects, and research contents, was established.

### 2.5 Data analysis

The data analysis included bibliometric analysis and visualization. Descriptive statistical analyses, including quantitative analysis of the article type, institution, and distribution of journals and keywords, were performed on the included articles. CiteSpace was used for institution collaboration network analysis and topic co-occurrence analysis. VOSviewer was used for author collaboration network analysis and keyword co-occurrence analysis. In VOSviewer, node size is positively correlated with the number of articles (Chen et al.,2023). The specific analysis process using CiteSpace was as follows. First, all included articles were converted to the Refworks format and imported into CiteSpace 6.2.R4. Then the software was configured: 1-year used for time slicing, “Author,” “Institution,” or “Keyword” selected as appropriate for node type, 50 set for Top N% per slice, and “pathfinder” and “pruning sliced networks” applied to prune and simplify the networks. Co-occurrence and clustering analysis were performed against Author, Institution, and Keyword, with the results visualized. The log-likelihood-ratio (LLR) algorithm was used for clustering analysis.

## 3 Results

### 3.1 General information

One hundred and eighty-seven articles were included in the study, including 145 (77.5%) in Chinese and 42 (22.5%) in English. The included articles were submitted by 926 authors from 28 provinces, cities, autonomous regions, or special administrative regions (SARs), including Beijing, Hebei, Shaanxi, and Macao SAR, as well as 383 scientific research institutions in the United States, South Korea, Thailand, and Pakistan. One hundred and forty Chinese articles (77.5%) were jointly submitted by 643 authors from 257 institutions and 42 English articles (22.5%) were jointly submitted by 392 authors from 152 institutions. There were 78 theoretical studies (41.7%), 63 clinical studies (33.7%), and 46 basic research studies (24.6%).

### 3.2 Number of publications and time distribution

**FIGURE 2:**
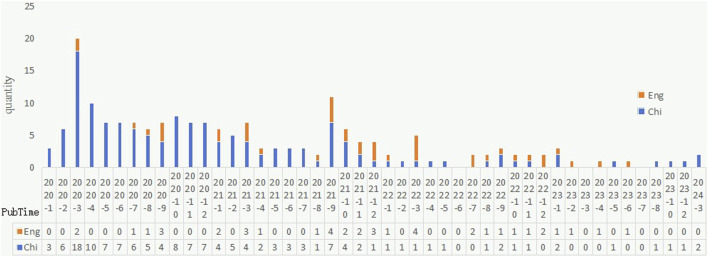
The trend of monthly publications.

The distribution of publication time for the 187 articles included in our study is shown in Figure 2. Ninety-three articles (49.7%) were published in 2020, 57 (30.5%) in 2021, 25 (13.4%) in 2022, 10 (5.3%) in 2023, and 2 (1.1%) in 2024. The articles for research on the prevention and treatment of COVID-19 with QFPDD predominated in the Chinese language. In addition, at the beginning of the COVID-19 outbreak, most articles were published in Chinese, and the number of publications reached a peak in March 2020. Since March 2020, the number of publications in Chinese generally showed a downward trend, with the number of English articles increasing first, then declining, and gradually becoming stable after August 2021.

### 3.3 Distribution of journals and highly cited articles

The 187 articles included in the study were published in 100 journals, including 73 (72.6%) Chinese language journals and 27 (28.4%) English language journals. Ten journals had four or more publications each (Supplementary Table S2), generating 67 articles (36.8%). For the Chinese journals, 19 (10.4%) articles were published in the *Journal of Traditional Chinese Medicine*, 8 (4.4%) were published in *China’s Naturopathy*, and 7 (3.9%) were published in *Pharmacology and Clinics of Chinese Materia Medica*. For the English journals, six (3.3%) articles were published in *Phytomedicine*. As of 31 April 2024, the mean number of citations of the 187 articles included in this study was 18.9 and the mean number of citations for Chinese articles was 16.5. The most frequently cited Chinese article, which was published in the *Journal of Traditional Chinese Medicine* in March 2020, was cited 226 times. The mean number of citations of the English articles was 27.4, and the most frequently cited article, which was published in *Pharmacological Research* in July 2020, was cited 156 times. In summary, the number of Chinese publications was much greater than the number of English publications, but the mean number of citations was higher for the English articles than for the Chinese articles. The five most frequently cited Chinese and English articles mainly focused on theoretical research and basic research on QFPDD.

### 3.4 Author collaboration network

**FIGURE 3:**
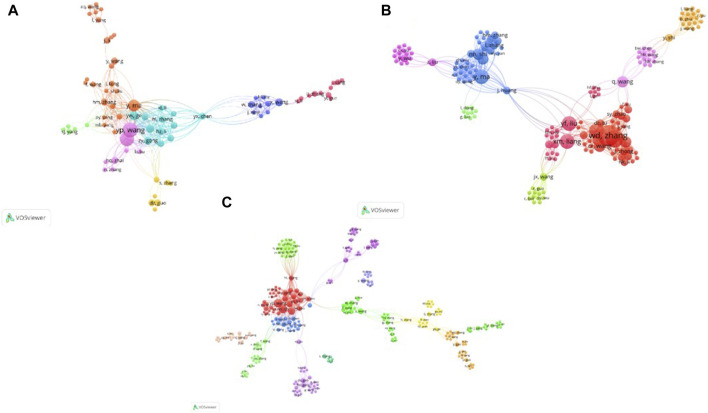
Co-occurrence diagram of authors in literatures. **(A)** Co-occurrence map of Chinese literature authors; **(B)** Co-occurrence map of English literature authors; **(C)**: Co-occurrence map of Chinese and English literature authors. Node size indicates the amount of text sent by the author; Internode links indicate cooperation among authors.

A total of 926 authors were involved in the 187 articles included in the study. According to Price’s law, the minimum number of articles published by core authors can be calculated using the formula m_*p*_ = 0.749 $\times \sqrt{n_{pmax}}$ (Zong, 2016). An m*p* value of 3.18 was obtained for the articles included in the present study. There were 31 core authors who published four or more articles. Wang Yanping from the Institute of Basic Research in Clinical Medicine, China Academy of Chinese Medical Sciences, was the author with the largest number of articles. A co-author network, in which the network scale referred to the total number of nodes included in the network and the number of links per node, was drawn using CiteSpace. The number of nodes and edges in the network determines its scale. Generally, a larger overall network scale indicates a more complex network structure. Each node represents an author and the thickness of the connecting lines indicates the intensity of the collaboration between authors. As shown in Figure 3A, the co-authorship network for Chinese articles had 643 nodes and 876 links, indicating that 643 authors jointly participated in the publication of the articles and cooperated with each other 876 times. This network shows research teams led by Wang Yanping and Ma Yan as the core. As shown in Figure 3B, the co-authorship network for English articles had 392 nodes and 1,176 links, indicating that 392 authors jointly participated in the publication of the articles and cooperated with each other 1,176 times. This network shows that research teams were led by Zhang Weidong and Ge Guangbo as the core. The co-authorship network for Chinese and English articles on the prevention and treatment of COVID-19 with QFPDD is shown in Figure 3C. There were 926 nodes and 2,412 links in the co-authorship network, indicating that 926 authors jointly participated in the publication of the articles and cooperated with each other 2,412 times. This network shows that two research teams were led by Wang Yanping and Zhang Weidong as the core.

According to the co-authorship networks, there were few isolated studies on the prevention and treatment of COVID-19 using QFPDD. The authors were interrelated, forming a research team with one scholar as the core. The core author nodes were large, indicating that the corresponding authors played key roles in relevant research on QFPDD. However, the overall structure of the network was relatively loose, indicating that there were multiple teams. The collaboration between different teams was weak, but the collaboration within each team was strong.

### 3.5 Collaboration between authors and institutions

**FIGURE 4:**
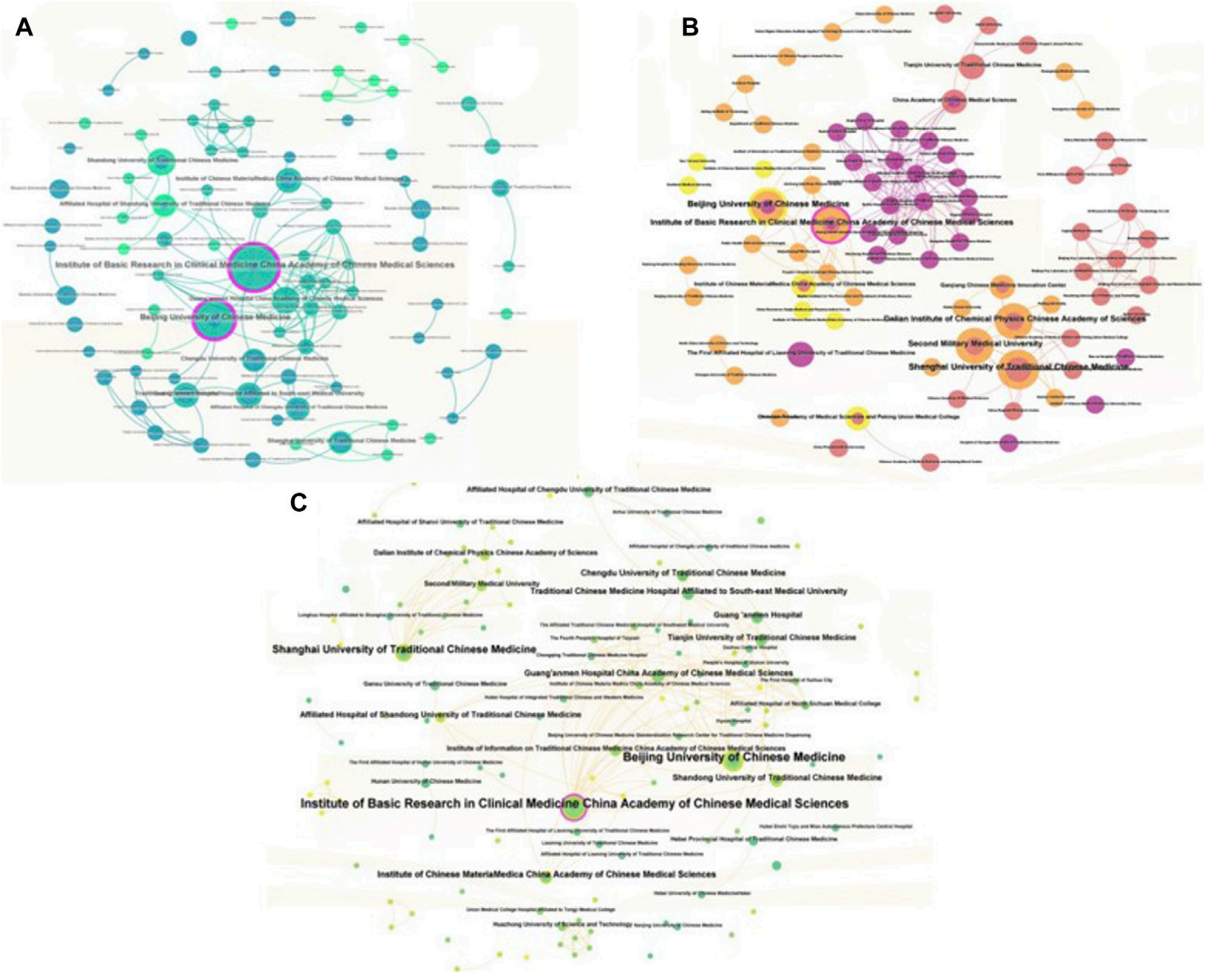
The collaboration knowledge map of literature institutions. **(A)** The collaboration knowledge map of Chinese literature institutions; **(B)** The collaboration knowledge map of English literature institutions; **(C)** The collaboration knowledge map of Chinese and English literature institutions. The node size indicates the amount of text sent by the organization. Internode links indicate inter-agency cooperation.

The 187 articles included in this study involved 383 scientific research institutions, with the Institute of Basic Research in Clinical Medicine, China Academy of Chinese Medical Sciences producing the largest number of articles (20). The Beijing University of Chinese Medicine (19); Guang’anmen Hospital, China Academy of Chinese Medical Sciences (12); the Institute of Chinese Materia Medica, China Academy of Chinese Medical Sciences (11); Shanghai University of Traditional Chinese Medicine (10); and the Affiliated Hospital (T.C.M) of Southwest Medical University (10) were also among the top 10 institutions most productive. As shown in Figure 4A, the co-occurrence network of institutions publishing Chinese articles had 257 nodes and 636 links, indicating that 257 scientific research institutions jointly participated in the publication of the articles and cooperated with each other 636 times. The network had the Institute of Basic Research in Clinical Medicine, China Academy of Chinese Medical Sciences and the Beijing University of Chinese Medicine as the core. As shown in Figure 4B, the co-occurrence network of institutions publishing English articles had 152 nodes and 456 links, indicating that 152 scientific research institutions jointly participated in the publication of the articles and cooperated with each other 456 times. The network had the Shanghai University of Traditional Chinese Medicine and the Institute of Basic Research in Clinical Medicine, China Academy of Chinese Medical Sciences as the core. The co-occurrence network of institutions publishing Chinese and English articles is shown in Figure 4C. The network showed 383 nodes and 1,139 links, indicating that 383 scientific research institutions jointly participated in the publication of the articles and cooperated with each other 1,139 timescollaboration. The network had the Institute of Basic Research in Clinical Medicine, China Academy of Chinese Medical Sciences and Beijing University of Chinese Medicine as the core.

### 3.6 Keyword co-occurrence and cluster analysis

**FIGURE 5:**
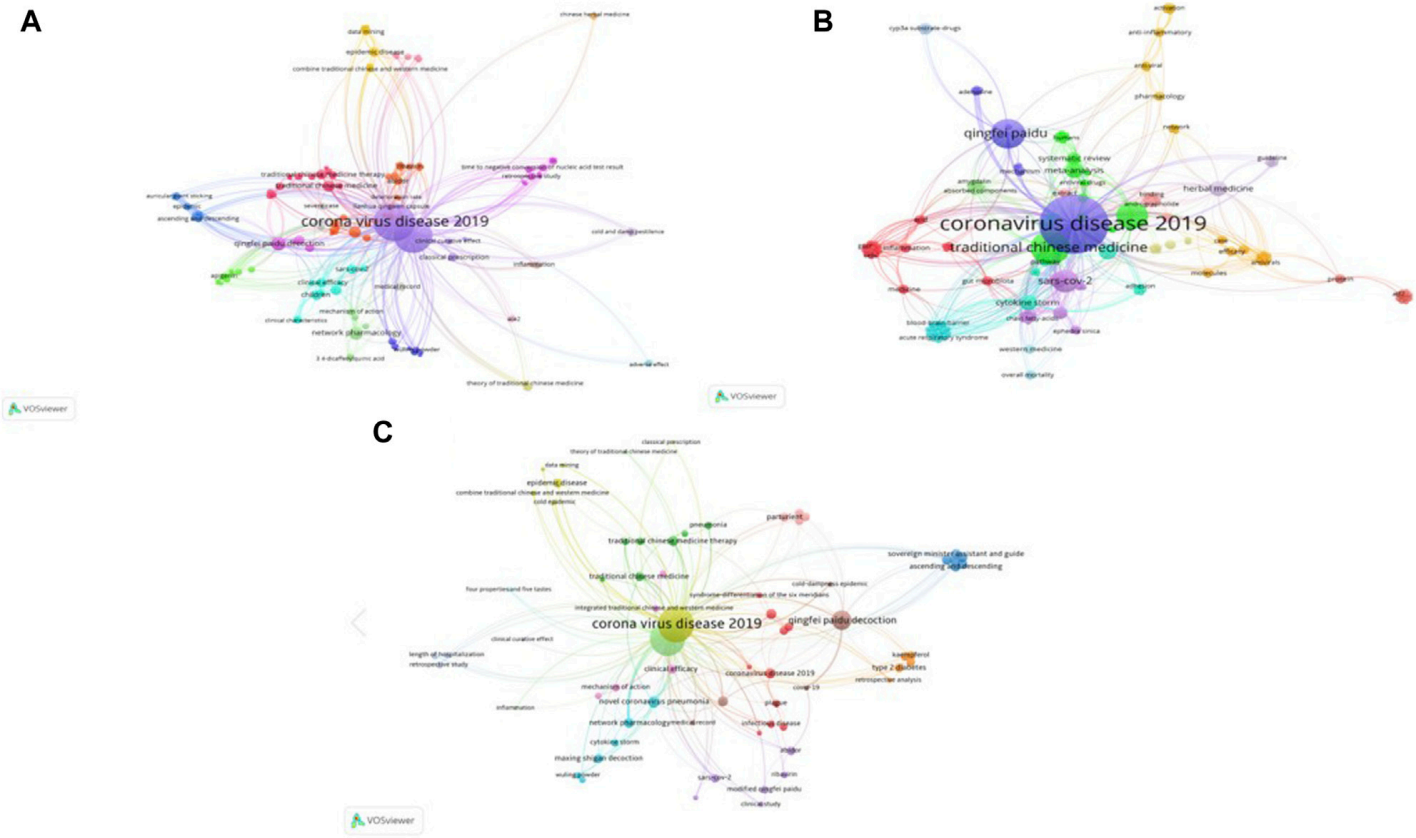
Co-occurrence diagram of keywords in literatures. **(A)** Co-occurrence diagram of keywords in Chinese literatures; **(B)** Co-occurrence diagram of keywords English literatures; **(C)** Co-occurrence diagram of keywords in Chinese and English literatures. The font size of keywords represents their frequency in the literature. Distances between items indicate correlation.

There were 412 keywords in the 187 articles, including 319 in the Chinese articles and 112 in the English articles. The co-occurrence network of English keywords included 112 nodes and 651 links. The number and content of the keywords varied according to the research topic. Keyword clustering is shown in Figures 5A–C. The keyword “corona virus disease 2019” occurred 175 times and “qingfei paidu decoetion” occurred 136 times. Other common keywords included “network pharmacology” (17 times), “traditional Chinese medicine” (16 times), “integrated Chinese and Western medicine” (8 times), “clinical efficacy” (8 times), “epidemic disease” (8 times), and “classical prescription” (6 times).

Cluster analysis is a statistical method for classifying data with multiple indicators. Cluster analysis of keywords included in the literature was used to explore the distribution of research hotspots on the use of QFPDD to prevent and treat COVID-19.

**FIGURE 6:**
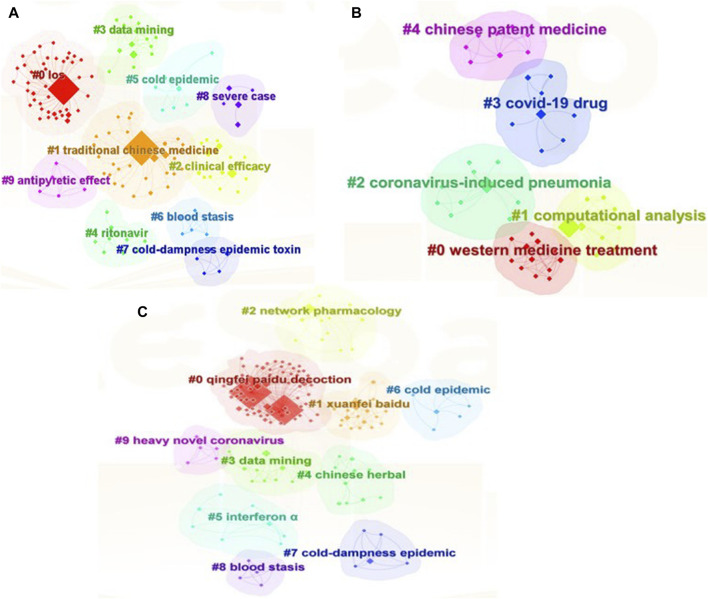
Keyword clustering network. **(A)** Keyword clustering network of Chinese literatures; **(B)** Keyword clustering network of English literatures; **(C)** Keyword clustering network of Chinese literatures English literatures.

In CiteSpace, the LLR algorithm was used to perform cluster analysis on the keywords in the literature. The cluster network of the Chinese literature is shown in Figure 6A. The clustering modularization (Q) value was 0.4425 (>0.4), indicating that the clustering was effective. The mean silhouette score (S) was 0.761 (>0.5), indicating that the network homogeneity was high and the keywords were closely related; thus, the clustering was significant and reasonable. Ten main clusters were formed by keywords from the Chinese literature, mainly involving the clinical efficacy of QFPDD, the pathogenesis of COVID-19, and TCM treatments. Among these clusters (Supplementary Table S3-1), clusters #0, length of stay; #2, clinical efficacy; and #9, antipyretic effect were mainly related to the study of the clinical efficacy of QFPDD. Outcomes such as hospitalization time, time to NAT negative conversion, antipyretic effect, adverse reactions, and laboratory indicators, were analyzed to explore the clinical efficacy of QFPDD. Cluster #1, traditional Chinese medicine; #5, cold epidemic; #6, blood stasis; and #7, cold-dampness epidemic toxin were mainly related to the study of the clinical characteristics and pathogenesis of COVID-19, as described in TCM. COVID-19 conforms to the characteristics of cold-dampness epidemic and belongs to the category of “epidemic disease” in TCM. The disease is located in the lungs and spleen, with cold-dampness damaging *yang* as the main line. It also has transformation syndromes, such as dissolving heat, changing to dryness, damaging *yin*, and leading to blood stasis. Cluster #4, ritonavir, and #8, severe case, were mainly related to the prevention and treatment of COVID-19 by integrating TCM with Western medicine, demonstrating the efficacy of integrating treatment with TCM as the core in preventing and treating COVID-19 for patients with mild symptoms and critically ill patients. Cluster #3, data mining, represents the study of QFPDD using data mining techniques.

The clustering network of the keywords from the English literature is shown in Figure 6B. A Q value of 0.442 (>0.4) and an S value of 0.7671 (>0.5), indicated that the clustering was effective and reasonable, and the results were credible. Five clusters were formed for English keywords (Supplementary Table S3-2). Cluster #0, Western medicine treatment, mainly related to systematic reviews and meta-analyses of QFPDD. Cluster #1, computational analysis; #2, coronavirus-induced pneumonia; and #3, COVID-19 drug were mainly related to the study of drug-metabolizing enzymes, drug interactions, and analyses by network pharmacology. Cluster #4, Chinese patent medicine, mainly referred to the study of TCM formulae and treatment methods of QFPDD for the prevention and treatment of COVID-19.

The clustering network obtained for the keywords in Chinese and English literature is shown in Figure 6C. A Q value of 0.4592 (>0.4) and an S value of 0.9218 (>0.5) indicated that the clustering was effective and reasonable, and the results were credible. Ten clusters were formed by Chinese and English keywords (Supplementary Table S3-3). Cluster #0, QFPDD; #2, network pharmacology; #9, heavy novel coronavirus; #10, severe case were related to clinical research and mechanistic studies focusing on QFPDD, including network pharmacology research, molecular docking studies, and studies of common adverse reactions and clinical indicators in patients with severe COVID-19. Cluster #1, Xuanfei Baidu; #4, Chinese herbal; and #5, interferon α, were related to the prevention and treatment of COVID-19. These keywords were used for article reporting of both traditional Chinese and Western medicine research focusing on α interferon, ribavirin, and arbidol; the three TCM drugs and four herbal formulas (3-drugs-4-formulas); ventilating lung *qi* and eliminating toxin; and resolving dampness and eliminating toxin for the prevention and treatment of COVID-19. Cluster #6, cold epidemic; #7, cold-dampness epidemic; and #8, blood stasis, summarize the important diagnostic criteria for COVID-19 and the pathogenesis of COVID-19, which is characterized by spleen dampness, lung dryness, and influenza. Blood stasis, *qi* deficiency, and *yin* deficiency belong to the category of cold-dampness epidemic. Cluster #3, data mining, represents the study of QFPDD using data mining techniques.

**FIGURE 7:**
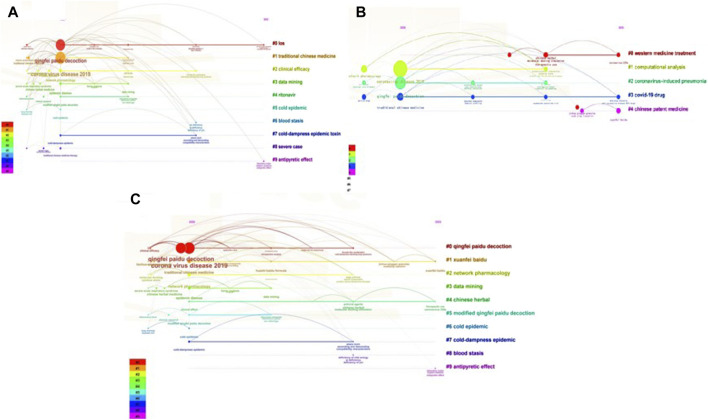
Time-line atlas of keyword clustering of literatures. **(A)** Time-line atlas of keyword clustering of Chinese literature; **(B)** Time-line atlas of keyword clustering of English literatures; **(C)** Time-line atlas of keyword clustering of Chinese literatures and English literatures.

In CiteSpace, the time span of each cluster and the association between different clusters were visually analyzed using a timeline. The keyword clustering timeline network is shown in Figure 7. Timeline Figure 7A shows that research on QFPDD was mainly reported in the Chinese literature in 2020, and covered theoretical research, basic research, method research, and other types of research. Chinese literature with cluster numbers #0, #2, and #7 had the longest time span from 2020 to 2024 indicating that the clinical study and clinical effects of QFPDD for the prevention and treatment of COVID-19 have raised considerable concerns. The time span for Clusters #2, #4, and #5 ended in 2021, indicating the decreased popularity of retrospective studies on QFPDD, patients with severe disease, and TCM symptom categories. Timeline Figure 7B shows that research published in English was conducted over a relatively evenly distribution time span. The longest time span was found for those in Cluster #3. Research on drugs used for the prevention and treatment of COVID-19 was performed over the longest period of time and also gained popularity recently. This trend has remained unchanged. Research on Cluster #4, traditional Chinese medicine, was concentrated in 2023, and included research on Jinhua Qinggan, Xuebijing, and Xuanfei Baidu, which belong to the four prescriptions in three TCM drugs and four herbal formulae category. Therefore, research on the use of TCM for the prevention and treatment of COVID-19 may emerge as a trending topic. Figure 7C shows the timeline of keyword clustering in the Chinese and English literature. Clusters #1 and #4 have a long-time span. TCM, which is dominated by three TCM drugs and four herbal formulae, has gained significant attention both domestically and internationally. This field may become a major research focus in the future. Research conducted on Clusters #1, #2, and #7 spanned from 2020 to 2022 and covered both clinical and basic studies on the prevention and treatment of COVID-19 using QFPDD. The majority of relevant research was conducted in 2020, and the popularity of this research topic began to decline in 2024.

### 3.7 Keyword emergence analysis

**FIGURE 8:**
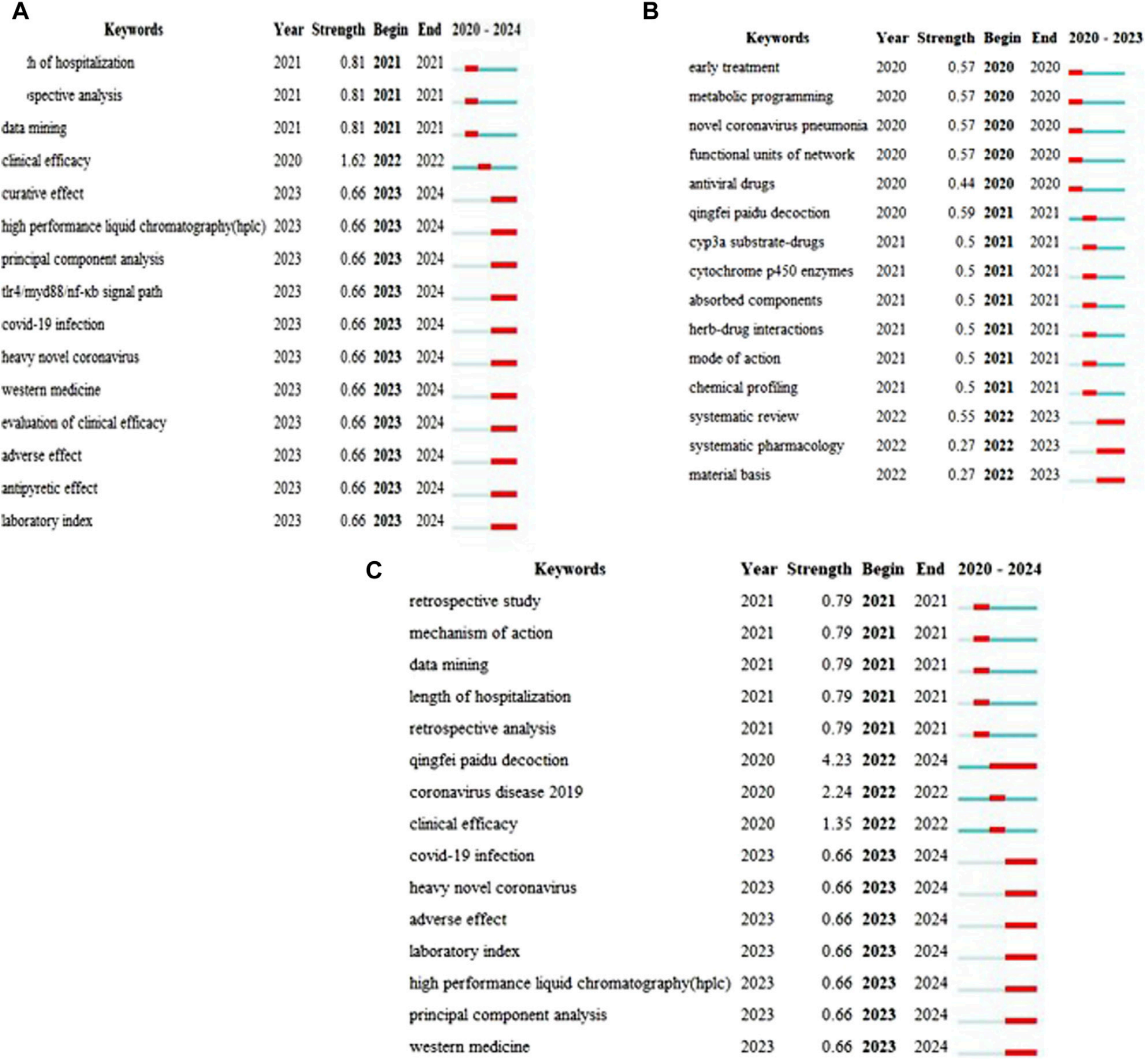
Keyword emergence diagram of literatures. **(A)** Keyword emergence diagram of Chinese literatures; **(B)** Keyword emergence diagram of English literatures; **(C)** Keyword emergence diagram of Chinese literatures and English literatures.

Keyword emergence refers to the significant increase in the frequency of occurrence of certain words in a short period of time. This phenomenon reflects the research content that researchers in the field were focused on during a particular period. By studying the rise and decline in hot research topics in recent years, we can predict the development direction of future research. Based on an analysis of the Chinese keyword emergence Figure 8A, the research field of QFPDD for the prevention and treatment of COVID-19 can be divided into two stages from 2020 to 2024. This research mainly focused on retrospective analyses from 2020 to 2021, and on evaluating the clinical efficacy of QFPDD for the prevention and treatment of COVID-19, the disease etiology and pathogenesis, and syndrome differentiation from 2022 to 2024. Based on an analysis of the English keyword emergence Figure 8B, the research field of QFPDD for the prevention and treatment of COVID-19 can be divided into three stages from 2020 to 2024. This research mainly focused on early treatment, antiviral drugs, and other related studies in 2020; the mechanism of action of QFPDD in the prevention and treatment of COVID-19 in 2021; and systematic reviews in 2022–2023. Based on an analysis of the Chinese and English keyword emergence Figure 8C, the research field of QFPDD for the prevention and treatment of COVID-19 can also be divided into three stages from 2020 to 2023. This research mainly focused on the use of TCM for the prevention and treatment of COVID-19 in 2020; retrospective studies of QFPDD for the prevention and treatment of COVID-19 in 2021; and clinical efficacy, etiology, and pathogenesis from 2022 to 2024.

## 4 Discussion

In this study, we systematically analyzed the number and time distribution of publications, the collaboration between authors and institutions, the co-occurrence of keywords, and cluster analysis of studies on the efficacy of QFPDD in the prevention and treatment of COVID-19. We also conducted metrological research and visual analysis to intuitively present the current research status, hotspots, and trends for the use of QFPDD in the prevention and treatment of COVID-19.

Chinese language articles accounted for a significant majority (77.5%) of the 187 articles that were analyzed from January 2020 to March 2024. During the initial phase of the epidemic, 20 articles (11.0%) were published in March 2020, reaching a peak. As time progressed, the number of English articles gradually increased. The three journals with the most articles were in Chinese, and seven of the articles in these journals were cited 50 times or more, compared with two articles in English. In addition, the vast majority of principal investigators and institutions were Chinese. This may be attributed to various factors, including the nature of QFPDD as a TCM, the global interest in TCM, linguistic advantages, and the fact that QFPDD had not been launched on the market at the beginning of the COVID-19 outbreak. Nevertheless, as more reports regarding the efficacy of QFPDD for the prevention and treatment of COVID-19 have been published, QFPDD has garnered global attention from scholars, including research teams from countries such as Canada, South Korea, and the United States. The South Korea Medical Association investigated the use of Chinese herbal medicine in 2,324 patients with COVID-19 from March 9 to 30 June 2020. China-originated QFPDD ranked first (13.4%) in the treatment of fever during the first dose, followed by improvements in headache, chills, expectoration, dry cough, sore throat, fatigue, muscle pain, runny nose, nasal congestion, dyspnea, chest tightness, diarrhea, and loss of appetite (Jang et al., 2021). Qingfei Paidu Granule was approved for marketing by the National Medical Products Administration on 02 March 2021 (National Health Commission of the PRC, 2020). The marketing of Qingfei Paidu Granule provides more options for global COVID-19 prevention and treatment, and also provides favorable conditions for the promotion of global multi-center research on Qingfei Paidu Granule in the future.

The author collaboration network showed that there were two main research teams led by Wang Yanping and Zhang Weidong as the core authors. The academic papers published with the team led by Wang Yanping as the core author mainly focused on multi-center clinical studies, while the team with Zhang Weidong as the core author mainly focused on basic research. This further reflects the geographical and institutional representativeness of the studies on QFPDD. All institutions were closely linked in the form of collaboration on research projects, forming an academic community centered on the core institutions of the Institute of Basic Research in Clinical Medicine, China Academy of Chinese Medical Sciences and the Institute of Interdisciplinary Integrative Medicine Research, Shanghai University of Traditional Chinese Medicine. The Institute of Basic Research in Clinical Medicine, China Academy of Chinese Medical Sciences had the largest number of clinical studies, with a total of 62 cooperative institutions, as well as collaboration between regional institutions, cross-regional collaboration, and close collaboration with high-yield institutions. Nevertheless, most of the current multi-center collaborations were with Chinese institutions and there was less collaboration on a global scale. Recently, Qingfei Paidu Granule has been approved for marketing as an over-the-counter drug in Canada, more than all indications approved in China, a new indication “influenza with the symptoms of above indications” has been added (Li, 2023). This marks a major step forward for TCM to gain global use and provides more support for future research to improve the evidence-based basis for its use at a higher level.

Keywords are usually one or more words or phrases that describe the subject of a paper. By analyzing keyword clustering, research hotspots and development trends in this field can be determined (Zheng et al., 2016). Therefore, keywords were retrieved to reflect the research hotspots on the prevention and treatment of COVID-19 with QFPDD. We found that the scale of clustering of basic research topics, such as inflammatory factors, targets, and network pharmacology was large, suggesting that in-depth studies on both *in vivo* and *in vitro* mechanisms should be carried out in the future to deeply analyze the antiviral and anti-inflammatory mechanisms of QFPDD in the treatment of COVID-19 infections, and provide experimental data support for guiding clinical medication. However, in terms of clinical studies, the clustering scale of keywords, such as clinical symptoms, exacerbation, time to NAT negative conversion, and adverse reactions was large, suggesting that multi-center prospective studies with large sample sizes should be conducted and efficacy evaluation should be carried out to provide more support for a high-level evidence-based basis for the use of QFPDD in the treatment of COVID-19.

## 5 Conclusion

In conclusion, we systematically and comprehensively analyzed the literature on the prevention and treatment of COVID-19 with QFPDD using CiteSpace and VOSviewer software, which intuitively showed the research status and current trends in this field. The results suggest that mechanistic studies and efficacy evaluations of QFPDD in the treatment of COVID-19 will be the focus and hotspot of future research. However, research hotspots can also constantly change over time, and the scope of research may be further expanded and extended. Researchers will apply new methods, tools, and technologies to strengthen the analysis of relevant research on QFPDD in the prevention and treatment of COVID-19 and promote the development of this area of research.

## Data Availability

The original contributions presented in the study are included in the article/Supplementary Material, further inquiries can be directed to the corresponding authors.

## References

[B1] Atlasi R. Ramezani A. Tabatabaei-Malazy O. Alatab S. Oveissi V. Larijani B. (2022). Scientometric assessment of scientific documents published in 2020 on herbal medicines used for COVID-19. J. Herb. Med. 35, 100588. 10.1016/j.hermed.2022.100588 35847990 PMC9272664

[B2] Chen J. Zhang Q. Liu X. Han Y. Gong Q. (2023). Knowledge mapping of COVID-19 and dentistry: a bibliometric analysis. Front. Public Health J10, 1040175. 10.3389/fpubh.2022.1040175 PMC9868823 36699914

[B3] Fan Y. P. Wang Y. P. Ma Y. Zhao C. Zhang H. M. (2020c). Analysis on composition mechanism of qingfei paidu decoction from pathogenesis of cold pestilence of COVID-19. Chin. J. Exp. Traditional Med. Formulae 26 (16), 1–5. 10.13422/j.cnki.syfjx.20201157

[B4] Fan Y. P. Wang Y. P. Zhang H. M. Wang Y. Y. (2020a). Analysis on the treatment of new coronavirus pneumonia (COVID-19) from the cold epidemic treatment. J. Traditional Chin. Med. 61 (5), 369–374. 10.13288/j.11-2166/r.2020.05.001

[B5] Fan Y. P. Zhang H. M. Wang Y. P. Lv C. Wang Y. Y. (2020b). A brief analysis of the attribute classification of coronavirus disease (COVID-19) in traditional Chinese medicine. J. J. Traditional Chin. Med. 61 (11), 921–927. 10.13288/j.11-2166/r.2020.11.001

[B6] Fu L. Shao S. Feng Y. Ye F. Sun X. Wang Q. (2021). Mechanism of microbial metabolite leupeptin in the treatment of COVID-19 by traditional Chinese medicine herbs. mBio 12 (5), e0222021. 10.1128/mBio.02220-21 34579576 PMC8546846

[B7] Ge Y. W. Zheng J. Zong X. Y. He R. Wang L. J. Zang G. D. (2022). Association between qingfei paidu granules initiated at different timepoints and clinical outcomes in asymptomatic COVID-19 omicron infection cases. J. Traditional Chin. Med. 63 (20), 1952–1957. 10.13288/j.11-2166/r.2022.20.010

[B8] Huang C. Su J. Xie X. Ye X. T. Li Z. Porter A. (2015). A bibliometric study of China’s science and technology policies:1949–2010. Scientometrics 102 (2), 1521–1539. 10.1007/s11192-014-1406-4

[B9] Jang S. Kim D. Yi E. Choi G. Song M. Lee E.-K. (2021). Telemedicine and the use of Korean medicine for patients with COVID-19 in South Korea: observational study. JMIR Public Health Surveillance 7 (1), e20236. 10.2196/20236 33342765 PMC7817255

[B10] Li C. B. Su Y. Liu Y. Q. Xue X. Gong H. X. Li T. T. (2020). Traditional Chinese medicine theory and modern pharmacology mechanism of qingfei paidu decoction in treating coronavirus disease 2019. J. Traditional Chin. Med. 61 (15), 1299–1302. 10.13288/j.11-2166/r.2020.15.003

[B11] Li N. (2023). Qingfei paidu granules approved for OTC marketing in Canada. J. Traditional Chin. Med. Manag. 31 (7), 30. 10.16690/j.cnki.1007-9203.2023.07.072

[B12] Li S. T. Wu H. Li Z. H. Peng B. Yang R. X. (2021). Expression of qingfei paidu formula treating COVID-19 according to classic formula theory. J. Chengdu Univ. TCM 44 (2), 11–14. 10.13593/j.cnki.51-1501/r.2021.02.011

[B13] Li T. Z. Xu G. G. (2020). Research updates of diagnosis and treatment of COVID-19. Med. J. Chin. People's Liberation Army 45 (3), 260–264. 10.11855/j.issn.0577-7402.2020.03.06

[B14] Ma Y. Zhu D. S. Chen R. B. Shi N. N. Liu S. H. Fan Y. P. (2020). Association of overlapped and un-overlapped comorbidities with COVID-19 severity and treatment outcomes: a retrospective cohort study from nine provinces in China. Biomed. Environ. Sci. 33 (12), 893–905. 10.3967/bes2020.123 33472729 PMC7817475

[B15] National Health Commission of the PRC, National Administration of Traditional Chinese Medicine (2020). Diagnosis and treatment plan for pneumonia caused by novel coronavirus infection(trial version 3). Tianjin J. Traditional Chin. Med. 37 (01), 1–3. 10.11656/j.issn.1672-1519.2020.01.01

[B16] Peng X. J. Yang X. J. Xu G. Chen H. B. Yang C. H. Gong W. L. (2020). Investigating clinical efficacy and mechanism of qingfei paidu decoction for treatment of COVID-19 based on integrative pharmacolog. J. Chin. J. Exp. Traditional Med. Formulae. 26 (16), 6–13. 10.13422/j.cnki.syfjx.20201638

[B17] Selva-Pareja L. Camí C. Roca J. Espart A. Campoy C. Botigué T. (2023). Knowledge, attitudes, and practices about COVID-19 pandemic: a bibliometric analysis. Front. Public Health 11, 1075729. 10.3389/fpubh.2023.1075729 37397778 PMC10313415

[B18] Shi N. N. Liu B. Liang N. Ma Y. Ge Y. W. Yi H. G. (2020). Association between early treatment with Qingfei Paidu decoction and favorable clinical outcomes in patients with COVID-19: a retrospective multicenter cohort study. Pharmacol. Res. 161, 105290. 10.1016/j.phrs.2020.105290 33181320 PMC7833425

[B19] Song B. Lei Y. Zhao L. H. Li X. Y. Shao J. Z. Yang Y. Y. (2020). Application of “Universal Formula”in the prevention and treatment of COVID-19 and its innovative development. J. Chin. J. New Drugs. 29 (16), 1807–1812. 10.3969/j.issn.1003-3734.2020.16.002

[B20] Sun Y. N. Lu W. L. Li H. Xiao Y. Yang L. Yang H. J. (2021). Qingfei paidu decoction for treatment of mild/moderate COVID-19 in 295 cases: a multi-centered. J. Traditional Chin. Med. 62 (7), 599–603. 10.13288/j.11-2166/r.2021.07.010

[B21] Tong L. Ma Y. Fan Y. P. Liu S. H. Zhang L. Zhang Y. (2021). The composition principle of qingfei paidu decoction and its application in epidemics based on literature review. J. Traditional Chin. Med. 62 (21), 1877–1881. 10.13288/j.11-2166/r.2021.21.007

[B22] Wang Q. Zhu H. Li M. Liu Y. Lai H. Yang Q. (2021). Efficacy and safety of qingfei paidu decoction for treating COVID-19: a systematic review and meta-analysis. Front. Pharmacol. 12, 1816–1826. 10.3389/fphar.2021.688857 PMC8387832 34456720

[B23] World Health Organization (2022). WHO expert meeting on evaluation of traditional Chinese medicine in the treatment of COVID-19. *E. coli* . Available at: https://www.who.int/publications/m/item/who-expert-meeting-on-evaluation-of-traditional-chinese-medicine-in-the-treatment-of-covid-19 (Accessed June 23, 2023).

[B24] World Health Organization (2024). WHO COVID-19 dashboard. Available at: https://data.who.int/dashboards/covid19/cases?n=c (Accessed May 17, 2024).

[B25] Xiong Y. Bao L. Ma Y. Zhang L. Qin C. Huang L. (2024). Wen-Yi and Chinese medicine: why we need to pay attention? Sci. Bull. 69, 1617–1622. 10.1016/j.scib.2024.03.059 38704357

[B26] Yu X. Y. Zhang S. Yan F. F. Su D. Z. (2020). Comparison of clinical efficacy of Qingfei Paidu decoction combined with western medicine in 43 cases and single western medicine in 46 cases in the treatment of COVID-19. J. Shandong Univ. Heal. Sci. 58 (12), 47–53. 10.6040/j.issn.1671-7554.0.2020.0870

[B27] Zhang L. Zheng X. Bai X. Wang Q. Chen B. Wang H. (2021). Association between use of Qingfei Paidu Tang and mortality in hospitalized patients with COVID-19: a national retrospective registry study. Phytomedicine 85, 153531. 10.1016/j.phymed.2021.153531 33799224 PMC7914374

[B28] Zhao M. Qin E. Q. Zhang D. W. Xu Z. Zhao P. Wang F. S. (2020). Expert consensus on clinical prevention and treatment of COVID-19. J. Med. J. Chin. People's Liberation Army 45 (11), 1109–1116. 10.11855/j.issn.0577-7402.2020.11.01

[B29] Zheng Y. N. Xu X. Y. Liu Z. H. (2016). Study on the method of identifying research fronts based on keywords Co-occurrence. Libr. Inf. Serv. 60 (4), 8592. 10.13266/j.issn.0252-3116.2016.04.012

[B30] Zong S. P. (2016). Evaluation of core authors based on Price law and the comprehensive index method: a case study of Chinese Journal of Scientific and Technical Periodicals. Chin. J. Sci. Tech. 27 (12), 1310–1314. 10.11946/cjstp.201610080819

[B31] Zong X. Y. Liang N. Wang J. Y. Li H. Wang D. Chen Y. (2022). Treatment effect of qingfei paidu decoction combined with conventional treatment on COVID-19 patients and other respiratory diseases: a multi-center retrospective case series. Front. Pharmacol. 13, 849598. 10.3389/fphar.2022.849598 35910390 PMC9326303

