# Supplementary material for: Status and hotspot analysis of Qingfei Paidu Decoction for the prevention and treatment of COVID-19 based on bibliometric analysis
Source: Front Pharmacol. 2024 Jul 31;15:1422773. doi: 10.3389/fphar.2024.1422773 (PMC11322107; doi:10.3389/fphar.2024.1422773)
Supplement: Supplementary file 2 [file Table2.DOCX]

Supplementary Material

Table 2 Top 10 Chinese and English Literature Periodicals

| Journal | Article number | Composition ratio |
| --- | --- | --- |
| Journal of Traditional Chinese Medicine | 19 | 10.2% |
| China's Naturopathy | 8 | 4.3% |
| Pharmacology and Clinics of Chinese Materia Medica | 8 | 4.3% |
| Phytomedicine | 6 | 3.2% |
| Acta Chinese Medicine | 6 | 3.2% |
| Frontiers in Pharmacology | 4 | 2.1% |
| Journal of Shandong University of Traditional Chinese Medicine | 4 | 2.1% |
| World Chinese Medicine | 4 | 2.1% |
| Chinese Traditional and Herbal Drugs | 4 | 2.1% |
