# Supplementary material for: Status and hotspot analysis of Qingfei Paidu Decoction for the prevention and treatment of COVID-19 based on bibliometric analysis
Source: Front Pharmacol. 2024 Jul 31;15:1422773. doi: 10.3389/fphar.2024.1422773 (PMC11322107; doi:10.3389/fphar.2024.1422773)
Supplement: Supplementary file 3 [file Table3.DOCX]

Supplementary Material

Table 3-1 Keyword clustering analysis of top 10 Chinese literatures

| Cluster name | Key words（LLR） |
| --- | --- |
| #0Lenth of Stay | Lenth of Stay (8.5, 0.005); traditional chinese medicine (6.91, 0.01); negative time of nucleic acid (6.36, 0.05); medical record (4.23, 0.05); lianhua qingwen capsule (4.23, 0.05) |
| #1traditional chinese medicine | traditional chinese medicine (16.6, 1.0E-4); classical prescription (6.16, 0.05); sars-cov-2 (4.11, 0.05); pathogenesis (4.1, 0.05); literature research (4.1, 0.05) |
| #2clinical efficacy | clinical efficacy (16.54, 1.0E-4); network pharmacology (10.96, 0.001); thunder-fire moxibustion (10.96, 0.001); cold-dampness blocking lung syndrome (10.96, 0.001); integrated pharmacology (5.44, 0.05) |
| #3data mining | data mining (12.58, 0.001); qingfei paidu decoction (6.68, 0.01); chinese herbal medicine (6.24, 0.05); frequency analysis (6.24, 0.05); middle east respiratory syndrome (6.24, 0.05) |
| #4ritonavir | ritonavir (7.79, 0.01); modified qingfei paidu decoction (7.79, 0.01); interferon (7.79, 0.01); chloroquine phosphate (7.79, 0.01); ribavirin (7.79, 0.01) |
| #5cold epidemic | cold epidemic (8.33, 0.005); spleen dampness (8.33, 0.005); dryness evil (8.33, 0.005); lung dryness (8.33, 0.005); composition mechanism (8.33, 0.005) |
| #6blood stasis | blood stasis (8.66, 0.005); qi deficiency (8.66, 0.005); yin deficiency (8.66, 0.005); parturient (8.66, 0.005); traditional chinese medicine (0.23, 1.0) |
| #7cold-dampness epidemic toxin | cold-dampness epidemic toxin (7.37, 0.01); compatibility characteristics (7.37, 0.01); ascending and descending (7.37, 0.01); epidemic (7.37, 0.01); stasis toxin (7.37, 0.01) |
| #8severe case | severe case (14.55, 0.001); traditional chinese medicine therapy (10.79, 0.005); integrated traditional chinese and western medicine (7.19, 0.01); experience case (7.19, 0.01); coronavirus infection (7.19, 0.01) |
| #9antipyretic effect | antipyretic effect (8.66, 0.005); heavy novel coronavirus (8.66, 0.005); western medicine (8.66, 0.005); adverse effect (8.66, 0.005); laboratory index (8.66, 0.005) |

Table 3-2 Keyword clustering analysis of top 5 English literatures

| Cluster name | Key words（LLR） |
| --- | --- |
| #0western medicine treatment | western medicine treatment (7.83, 0.01); systematic review (7.83, 0.01); meta-analysis (7.83, 0.01); effect (5.1, 0.05); pai-du decoction (0.5, 0.5) |
| #1computational analysis | computational analysis (8.94, 0.005); mild tosevere stage (8.94, 0.005); covid-19 patient (8.94, 0.005); qingfei paidu decoctionin (8.94, 0.005); computer-aided drug design (7.64, 0.01) |
| #2coronavirus-induced pneumonia | coronavirus-induced pneumonia (11, 0.001); herb-drug interaction (8.59, 0.005); drug-metabolizing enzyme (8.59, 0.005); covid-19 injury (7.97, 0.005); metabolic programming (7.97, 0.005) |
| #3covid-19 drug | covid-19 drug (11.11, 0.001); drug interaction (11.11, 0.001); traditional chinese medicine (5.81, 0.05); mice serum (5.49, 0.05); tissue (5.49, 0.05) |
| #4chinese patent medicine | chinese patent medicine (7.43, 0.01); infection mechanism (7.43, 0.01); chinese medicine formula (7.43, 0.01); promising therapeutic approaches (7.43, 0.01); potential target (7.43, 0.01) |

Table 3-3 Keyword clustering analysis of top11Chinese and English literatures

| Cluster name | Key words（LLR） |
| --- | --- |
| #0qingfei paidu decoction | qingfei paidu decoction (11.67, 0.001); corona virus disease 2019 (10.48, 0.005); clinical efficacy (5.45, 0.05); mechanism of action (5.45, 0.05); los (5.45, 0.05) |
| #1xuanfei baidu | xuanfei baidu (8.58, 0.005); huashi baidu formula (8.58, 0.005); traditional chinese medicine (8.44, 0.005); corona virus disease 2019 (7.45, 0.01); hrms (4.27, 0.05) |
| #2network pharmacology | network pharmacology (15.67, 1.0E-4); cytokine storm (10.16, 0.005); molecular docking (10.16, 0.005); qingfei paidu decoction (5.16, 0.05); molecules (5.05, 0.05) |
| #3data mining | data mining (12.82, 0.001); chinese herbal medicine (6.36, 0.05); middle east respiratory syndrome (6.36, 0.05); cluster analysis (6.36, 0.05); factor analysis (6.36, 0.05) |
| #4chinese herbal | chinese herbal (11.51, 0.001); therapeutic use (11.51, 0.001); humans (7.8, 0.01); corona virus disease 2019 (6.39, 0.05); coronavirus disease 2019 drug treatment (5.71, 0.05) |
| #5interferon α | interferon α (7.91, 0.005); ribavirin (7.91, 0.005); abidor (7.91, 0.005); modified qingfei paidu decoction (7.91, 0.005); chloroquine phosphate (7.91, 0.005) |
| #6cold epidemic | cold epidemic (8.45, 0.005); spleen dampness (8.45, 0.005); dryness evil (8.45, 0.005); lung dryness (8.45, 0.005); composition mechanism (8.45, 0.005) |
| #7cold-dampness epidemic | cold-dampness epidemic (7.49, 0.01); compatibility characteristics (7.49, 0.01); ascending and descending (7.49, 0.01); cold-dampness epidemic toxin (7.49, 0.01); epidemic (7.49, 0.01) |
| #8blood stasis | blood stasis (8.79, 0.005); qi deficiency (8.79, 0.005); yin deficiency (8.79, 0.005); parturient (8.79, 0.005); coronavirus disease 2019 (0.39, 1.0) |
| #9heavy novel coronavirus | heavy novel coronavirus (8.79, 0.005); western medicine (8.79, 0.005); antipyretic effect (8.79, 0.005); adverse effect (8.79, 0.005); laboratory index (8.79, 0.005) |
| #10severe case | severe case (16.07, 1.0E-4); integrated traditional chinese and western medicine (7.91, 0.005); traditional chinese medicine therapy (7.91, 0.005); experience case (7.91, 0.005); diagnosis and treatment plan (7.91, 0.005) |
